# Hijab Pornography: A Content Analysis of Internet Pornographic Videos

**DOI:** 10.1177/10778012211021125

**Published:** 2021-06-22

**Authors:** Yaser Mirzaei, Somayyeh Zare, Todd G. Morrison

**Affiliations:** 1 University of Saskatchewan, Saskatoon, Canada

**Keywords:** hijab pornography, content analysis, aggression, objectification, exploitation, agency

## Abstract

A content analysis was conducted to explore sexual indicators of aggression, objectification, exploitation, and agency in 50 “hijab” pornographic videos. Our findings suggest that women were the target of aggressive acts in all videos, with gagging (42%) and spanking (38%) being the most common. Also, in comparison with men, women were more likely to be objectified and exploited, and less likely to possess agency. Limitations of the current study and directions for future research are detailed.

Internet pornography is omnipresent. It comprised approximately 12% of all internet websites in 2009 (i.e., 4.2 million sites; DeKeseredy & Corsianos, 2015; Twohig et al., 2009). In the same year, there were “68 million daily pornographic search engine requests,” which accounted for 25% of total requests recorded (DeKeseredy & Corsianos, 2015, p. 1). Since 2009, the popularity of online pornography has skyrocketed. For example, PornHub.com reported that 41 billion individuals visited this site in 2019 (i.e., around 115 million daily visits; DeKeseredy, 2020). According to TechRadar.com, pornographic websites are among the most well trafficked worldwide; XVideos.com and PornHub.com are the most popular ones, with an average of 3.14 and 2.85 trillion visits per month, respectively. To compare, Amazon and Netflix were visited monthly 2.9 and 2.21 trillion times per month, respectively (Khalili, 2020). Moreover, the proportion of individuals watching pornographic material on the internet has increased appreciably over the past two decades. In 2005, a sizable percentage of individuals, especially men, viewed pornographic videos on internet webpages (e.g., a U.S. national survey revealed that 4% of women and 25% of men reported viewing a pornographic website during the month prior to participating in the study; Buzzell, 2005). More recently, in 2020, 1,392 adults residing in the United States were surveyed through Amazon Mechanical Turk, with results indicating that 91.5% of men and 60.2% of women reported consuming pornography^1^ in the past month (Solano et al., 2020). The popularity of online pornography may be attributed to the fact that much of it is free. To illustrate, Wondracek et al. (2010) examined 700 pornographic sites (i.e., 270,000 URLs on more than 35,000 domains) and found that 91.9% of them were free.

Despite the availability of free online content, the pornography industry generates massive revenue (e.g., in 2010, the figures were approximately US$13 billion in the United States and US$100 billion globally; Rosen, 2013). The profitability and widespread consumption of pornography have resulted in this medium triggering considerable interest among social scientists. Some of the questions that researchers have attempted to address include the following: (a) What effects does pornography use have on people’s attitudes toward sexuality and sexual behaviors? and (b) Does the need for greater profitability result in pornography producers creating more “extreme” content?

Prior to examining the aforementioned questions, it is critical that a definition of pornography be furnished. Researchers have defined pornography in multitudinous ways (Rea, 2001; Rose, 2013); however, common features may be identified. Many definitions, for example, emphasize that pornography depicts and increases violence against women (DeKeseredy, 2020; Foubert, 2016; Weitzer, 2011), promulgates negative attitudes toward women (Golde et al., 2000), objectifies women’s bodies (Brecher, 2015), and is rife with degrading acts (Gorman et al., 2010) as well as racist depictions (Miller-Young, 2014; Zheng, 2017). However, these sorts of definitions are value-laden and use terms that are difficult to operationalize (e.g., degradation^2^). Furthermore, the use of definitions that clearly reflect an advocacy position may result in findings being trivialized or dismissed. Thus, to avoid these pitfalls, we opted for a broader and ostensibly more neutral definition. Specifically, to paraphrase Rose (2013), the term *pornography* refers to materials that, for its intended audience, would be considered sexually arousing. This definition does not presume that said material is heterosexual, nor does it preclude the possibility of pornographic material having a sex-positive feminist orientation.

It is vital to note that pornography is not a monolithic medium; rather, it is a superordinate category, which encompasses myriad niche forms. One niche form that has emerged recently and garnered attention is hijab pornography (i.e., pornographic material that portrays at least one of the female performers as wearing Islamic hijab and, in so doing, accentuates Muslim women’s culturally specific way of dressing). For example, in Germany, between January 2015 and April 2018, online search requests for keywords related to hijab pornography such as refugee porn increased by 114%. In Hungary, during the summer of 2015, the number of germane search requests increased 151% by September 2015. In Austria, the number of searches increased by 195% in December 2017. In Poland, interest in “hijab porn” surged by 207% during November 2015 (Amjahid, 2018).

Despite consumers’ apparent interest in hijab pornography, we could find no empirical literature that was relevant to the objectives of the current study (i.e., to investigate, using a standard content analysis, the types of messages about Muslim women’s sexuality that are disseminated by hijab pornography). To fill such a gap, we think the current study is necessary because its results would reveal, for example, the kinds of messages that hijab pornography seems to be producing. Also, it is important, as has been addressed so far in several published content analyses on pornography (e.g., Fritz & Paul, 2017; Peters et al., 2014; Vannier et al., 2014), that different genres and niche forms of pornography, especially emerging ones such as hijab pornography, are examined and compared. We believe that our study’s results will generate necessary but absent data that make us able to compare hijab pornography with other well-studied niche forms (e.g., “MILF” category; Vannier et al., 2014). Such comparisons would show, for example, if hijab pornographic videos (HPVs) portray more violence against women than do other categories (e.g., “MILF”).

Before outlining the theoretical framework of our study (i.e., Sexual Script Theory [SST]), we begin by explaining why we distinguish hijab pornography from race porn. Then, we offer a brief overview of existing content analyses of pornography, paying particular attention to the variables favored by researchers in this area (e.g., violence, objectification, exploitation, and agency). Furthermore, we detail SST and its applicability to the medium of pornography. We conclude by articulating the central research questions of the current study.

## Hijab Pornography Versus Race Porn

It should be noted that we view hijab pornography as distinct from race porn for a number of reasons. First, hijab *is* a specific way of dressing^3^ and *is* an indicator of Islam as a religion^4^ (i.e., Islam is *not* a race or an ethnicity; Adebayo, 2021; Dagli, 2020; Pratt, 2015). Similarly, Muslim is neither a racial nor an ethnic category; rather, it denotes a diverse group of people, situated throughout the world, who follow Islam (Dagli, 2020). The word “hijab” is an Arabic and Quranic (see Note 4; for example, Surah Al-Noor, Verse 31; Surah Al-Ahzab, Verse 59) term. When used as an adjective in relation to pornography, “hijab” refers to a specific code-set of dressing (Adebayo, 2021; Pratt, 2015) that highlights “religion” and *not* “race” (Dagli, 2020). Indeed, as we found in our content analysis of HPVs, female performers appearing in these videos were from a variety of races/ethnicities. For example, in HPVs produced in Eastern Europe, especially in the Czech Republic (e.g., czechsexcasting.com), female performers are predominantly White. In HPVs produced in North America, female performers’ races are more diverse. Indeed, we found examples where popular White porn stars wore hijab.^5^ Second, given that hijab is a religious, rather than racial, symbol, it would be most accurate to classify hijab pornography as a niche form of *religious* porn (e.g., nuns or Mormons).^6^ Third, the distinctions that we observe reflect the lived experience of two of the co-authors; both are from Iran and recognize the distinctions among terms that Westerners may conflate such as Muslim, Arab, and Islam.

## Previous Content Analyses of Pornography

Recent content analyses on pornographic videos have studied aggression, objectification, exploitation, and agency. For example, Klaassen and Peter (2015), in a content analysis of 305 professional popular pornographic internet videos, found that: (a) women were more often objectified through the absence of instrumentality (e.g., orgasms; 77.7% of men vs. 18.04% of women), whereas men were more objectified through dehumanization (e.g., close-up of face; 13.4% of men vs. 65.2% of women); (b) 28.2% of the videos showed men and women in hierarchal positions and the rest showed nonsignificant differences; (c) women were more submissive than men (40% vs. 12.1%) during sexual encounters; and (d) among violent acts, spanking and gagging were most frequent (30% and 22%, respectively). In another study of 100 free popular internet “Teen” and “MILF” videos, the researchers reported that: (a) fellatio and vaginal intercourse were the most frequent sexual acts performed (86% and 88%, respectively); (b) men and women did not differ in their control of the pace/direction of sexual activity, initiation of sexual activity, sexual experience, and professional status; and (c) although levels of exploitation for men and women did not differ significantly, women were more exploited than men (15% vs. 4%; Vannier et al., 2014). Moreover, by analyzing 100 *Mainstream* pornographic scenes, Fritz and Paul (2017) found that the focus on genitalia and physical aggression were significantly higher for women than men (82% vs. 68% and 36% vs. 1%, respectively). Female orgasm occurred in 15% of the scenes reviewed, whereas for men, the proportion was 61%. As well, men seemed to more frequently direct the sexual acts than did women (46% vs. 33%). Zhou and Paul (2016) studied 3,132 online pornographic videos and found that female-to-male oral stimulation was more common than male-to-female oral stimulation (51.5% vs. 18.3%, respectively). Men and women equally initiated sex acts. In 88% of the videos, women were the target of aggression. Furthermore, Gorman et al. (2010) content analyzed 45 internet pornographic videos and reported that: (a) fellatio (79%) and vaginal intercourse (68%) were the most frequent acts performed; (b) male-to-female genital stimulation occurred in 13% of the videos; (c) in 33% of the videos, the male performer was shown in a dominant position; (d) in 47% of the videos, the female performer was portrayed in a submissive role; and (e) cum-shots were included in 45% of the videos. By studying 304 scenes, Bridges et al. (2010) reported that: (a) 69% of aggression was committed by male performers toward female performers; (b) spanking, gagging, and insulting were the most common aggressive acts (75.3%, 53.9%, and 48.7%, respectively); (c) fellatio and vaginal intercourse were shown in 90.1% and 86.2% of the videos; and (d) male-to-female oral sex was portrayed in 53.9% of the scenes. Finally, Fritz et al. (2020) analyzed 4,009 heterosexual scenes from two websites and found that: (a) 45% of PornHub and 35% of XVideos scenes, respectively, included at least one aggressive act; (b) in 97% of the scenes, women were the target of violence (also, in 76% of the scenes, men were the perpetrators of violence); and (c) spanking, slapping, and gagging were the most common forms of physical aggression (32.1%, 12.2%, and 11.7%, respectively).

## SST

This theory has been widely employed in studies of pornography (e.g., Wright, 2012), especially in content analyses (e.g., Zhou & Paul, 2016). Simply stated, social scripts are “the mental representations individuals construct and then use to make sense of their experience” (Wiederman, 2015, p. 7). The basic premise of this theory is that all social behaviors are socially scripted (Simon, 2017) and change over time (McCormick, 2010; Monto & Carey, 2014). The more a person is exposed to a specific media script, the more likely the individual is to internalize and adopt the script as their worldview and, subsequently, behave in accordance with the tenets of that script (Sun et al., 2016). Moreover, different categories of pornography provide different sexual scripts. To illustrate, in their study, Fritz and Paul (2017) found that in *Mainstream* pornography (i.e., porn videos produced for mass consumption, which are easily accessible and free, and target male consumers; Fritz & Paul, 2017), 83% of scenes that featured the male performer having an orgasm concluded with an external cum-shot, in which ejaculation took place outside of a woman’s body. In *For Women* pornography (i.e., porn videos specifically directed at female consumers; Fritz & Paul, 2017), the proportion was 49%, and in *Feminist* pornography (i.e., videos written or produced by women containing portrayals of “genuine” female pleasure and empowerment; Fritz & Paul, 2017), the proportion was even smaller (36%). Consumers of *Mainstream* videos may internalize the message that sex culminates with male orgasm and that male ejaculation is of paramount importance to a satisfying sexual encounter, whereas persons viewing *For Women* or *Feminist* pornography may regard female sexual pleasure as more central.

Researchers also contend that the sexual scripts evident in pornography may influence viewers’ sexual behaviors (Gwinn et al., 2013; Weitzer, 2011; Wright, 2011) in different ways, for example, by conveying messages regarding: (a) what behaviors constitute sex, (b) what should or should not occur during a sexual encounter, (c) the anticipated consequences of a given sexual episode (Willis et al., 2020), and (d) revealing how pornography consumption might encourage violence against women in viewers (DeKeseredy, 2020; DeKeseredy & Hall-Sanchez, 2017 ; Foubert & Bridges, 2017).

## Statement of Purpose

This study is an exploratory content analysis in which the central aim is to investigate popular and well-studied indicators of aggression, objectification, exploitation, and agency in a sample of free online HPVs.

## Method

Sources focusing on content analysis (e.g., Krippendorff, 2018; Neuendorf, 2016) as well as several papers that employed this method (e.g., Bryman, 2016; Drisko & Maschi, 2015; Prior, 2014) served as “guides.” Also taken into consideration were highly cited content analyses conducted on pornography (e.g., Shor & Seida, 2019; Vannier et al., 2014).

### Sample^7^ and Inclusion Criteria

Many highly viewed pornographic websites are hubs for distributing other sites’ videos (Downing et al., 2014; for example, according to Alexa.com, XVideos has 4,806 total linking sites). Thus, we focused on the first five websites that appeared when searching for “hijab porn” on Google.com: PornHub, XVideos, Youporn.com, XHamster.com, and Xnxx.com (Peters et al., 2014; Shor & Golriz, 2019). These websites are among the top pornography sites listed on Alexa (e.g., XVideos ranks number 1; March 4, 2020). PornHub and YouPorn are affiliated with the “PornHub Network” owned by parent company Manwin Holding SARL (Wallace, 2011). Therefore, to minimize the possibility that the same video may be coded more than once, we retained YouPorn because its rank was higher than PornHub, based on Alexa’s 50 top porn websites as of March 4, 2020. Furthermore, by looking for “hijab porn” on each website, we sorted the videos based on popularity (i.e., the number of times the video was viewed) and then picked the eligible videos from the first 10 pages of XVideos from which 25 eligible videos were collected (range of viewership: 1,111,900–43,531,214 times; May 14, 2020). As YouPorn did not provide the mechanism to order videos of each category based on popularity, we searched for “hijab porn” on this site, and then gathered those videos on the first 10 pages that met the inclusion criteria, provided they had not been already selected from XVideos. This process was repeated for Xnxx and XHamster,^8^ respectively.

A video was deemed suitable for inclusion if it (a) depicted a female performer wearing hijab, (b) was professional (i.e., it would be included if it *seemed* “professional” regardless of whether it was tagged or titled as amateur), (c) included performers engaging in heterosexual sexual activity, (d) was longer than 5 min, (e) portrayed at least two individuals engaging in a sexual act (i.e., was not a solo performance), and (f) had been viewed more than 1 million times (Peters et al., 2014). Fifty videos were sampled from the four sites. In the rare event that a video contained multiple scenes (*k* = 1), only the first one was analyzed because it was the one most likely to be watched (Klaassen & Peter, 2015).

### Coders and Coding Process

The videos were watched and coded by one primary coder; however, to gauge interrater reliability (i.e., Kappa; McHugh, 2012), two additional coders were further employed. A code sheet of the four categories (e.g., objectification) as well as indicators (e.g., cum-shot) was designed to help the raters through the procedure (Morrison & Halton, 2009). Categories of interest, which were generated from the extant research, are detailed below.

### Indicators of Aggression

In studies on pornography, aggression is usually defined as physically (e.g., chocking) and/or verbally (e.g., insulting) violent acts. Based on the current literature (Bridges et al., 2010; Gorman et al., 2010; Klaassen & Peter, 2015), some of the indicators of aggression extracted include spanking (i.e., slapping on the buttocks as a punishment), slapping (i.e., hitting with the palm of one’s hand or a flat object), punching (i.e., striking with the fist), gagging (i.e., penis being thrust deeply into the mouth to make the person gag or even vomit), pushing roughly, pulling one’s hair roughly, choking (i.e., causing someone to stop breathing by squeezing the throat), confining (i.e., any act restraining one’s movements, for example, bondage and tying someone up or keeping one in a particular position to stop them from moving), torturing (i.e., any specific kind of torture, for example, waterboarding; mutilating, that is, inflicting a violent and disfiguring injury or serious damage), whipping (i.e., beating someone with a whip or similar instrument), using a weapon, verbal threats (e.g., a male security officer tells a female shoplifter that he will call the police if she does not agree to have sex with him), name calling and/or insulting (e.g., terms such as bitch, nasty, slut), and hate speech (e.g., insulting one’s race or religion).

### Indicators of Objectification

Objectification in pornographic materials can be defined as portraying one part of the body instead of a whole, complete human being (e.g., Loughnan et al., 2010; Morrison & Halton, 2009); for example, by filming the vagina or penis in close-up, these organs are objectified. Objectification can be further partitioned into two categories: (a) instrumentality, and (b) dehumanization. The former may be defined as using someone’s body parts for another person’s sexual pleasure (e.g., close-ups of breasts and vagina for the erotic gratification of the viewer). Dehumanization refers to concentrating on body parts, thereby denying other human characteristics such as feelings or thoughts (Klaassen & Peter, 2015). In the current study, some of the indicators of objectification generated from the available literature are as follows: (a) focus on and/or close up of body parts (e.g., breasts, buttocks) and/or face; (b) any combination of anal, vaginal, and/or oral penetration (e.g., one male performer penetrates the female performer’s anus, while another male performer penetrates her mouth; Gorman et al., 2010); (c) manual and/or oral stimulation of more than one man simultaneously (Peter & Valkenburg, 2009); (d) cum-shot (i.e., the male performer ejaculates on the woman with the semen being obvious); (e) cream-pie (i.e., the male performer ejaculates inside the female performer’s vagina or anus, with the semen being filmed oozing out); (f) fellatio (i.e., a woman using mouth, lips, tongue, and throat to orally stimulate a man’s penis); (g) gaping (i.e., keeping the anus wide open for a period of time to make it more flexible for inserting objects; Fritz & Paul, 2017); and (h) ass-to-mouth (i.e., withdrawal of the penis from the anus followed by immediate insertion into the mouth; Bridges et al., 2016).

### Indicators of Exploitation

Gorman et al. (2010) put indicators in the “exploitation” category if one or more performers are being used by another one. Based on the extant literature, five criteria were selected to determine the exploitation: (a) nonconsensual sex (e.g., forcing one into sexual activity; showing unwillingness while engaging in a sexual encounter), (b) hierarchy of status (i.e., one performer is depicted as occupying a lesser status than another performer, for example, housekeeper vs. homeowner, tenant vs. landlord), (c) age gap (the criterion is 20 years in the current study), (d) any dishonest/underhanded way of getting someone to have sex (e.g., getting someone intoxicated or drunk, and then having sex with them), and (e) having sex in exchange for drugs, food, shelter, money, protection, or employment (e.g., a male performer tells a female performer that she can have food if she agrees to have sex with him; Vannier et al., 2014).

### Indicators of Agency

Popular indicators appearing in content analyses of pornography (Bridges et al., 2010; Fritz & Paul, 2017; Gorman et al., 2010; Klaassen & Peter, 2015; Vannier et al., 2014) were selected: (a) reciprocity (i.e., both male and female orgasms are shown), (b) sex for own pleasure (i.e., both performers show pleasure, verbally and nonverbally, during the scene; self-touch), (c) stimulation of female genitals using tongue or lips while she is the focus of pleasure (e.g., cunnilingus or female anilingus), (d) initiating the sexual encounter (i.e., using verbal and/or nonverbal cues indicating willingness to start sexual activity), (e) dominant/submissive roles (e.g., traditional top vs. bottom positions are reversed; both male and female performers control the direction and/or pace of sex), and (f) sexual experience (i.e., female and male performers are being shown as equally experienced, for example, a performer would not be considered agentic if they are implicitly and/or explicitly depicted as inexperienced at doing a specific sexual activity).

## Results

Fifty videos were coded: 42 by the primary coder and eight videos by two other coders (three and five videos, respectively). The coders were from differing age, gender, racial, and educational backgrounds (e.g., two—one male and one female—were born and brought up in a Muslim culture). Using previous content analyses as a guide, a coding sheet that was divided into various categories (e.g., sexual acts, physical setting, and duration of the video) was developed to facilitate the content analysis. In terms of interrater reliability, on two occasions, Raters 1 and 2 and Raters 1 and 3 reviewed a small selection of the videos to: (a) determine whether the coding sheet was “user-friendly,” (b) establish a set of procedural coding rules, and (c) facilitate an initial estimate of consistency between coders. No discrepancies were noticed between the coding of Raters 1 and 2 and Raters 1 and 3.

### Descriptive Characteristics

The length of the 50 videos collected ranged from 5.05–40.27 min (*M* = 10.12, *SD* = 5.45). All the scenes, except for two (4%), portrayed the sexual encounters in a private setting (e.g., home or office). In 96% of the videos, the female performer kept her hijab (at least headscarf or burqa) on for the entire scene. Moreover, except for one video in which the male actor wore dishdasha or thobe (i.e., an ankle-length Arabian garment) and kaffiyeh (i.e., a traditional Arabian headdress), in the remainder (98%), no implicit or explicit reference to the male performer’s race, religion, or nationality was observed.

### Aggression in Hijab Pornography

The frequency of aggressive acts is illustrated in Table 1. Our results indicate that, in all of the videos we analyzed, only women were the targets of aggression. Gagging and spanking happened most frequently (42% and 38%, respectively). Other violent acts that were common included confining (20% of the videos), insulting (22% of the videos; for example, “whore”), and pushing (24% of the videos). The least common violent acts were slapping (14%), verbal threatening (10%; for example, a husband threatened his wife that he would whip her again if she continued to cheat on him), chocking (10%), hate speech (6%; for example, “Muslin bitch”), torturing (2%; that is, waterboarding), and whipping (2%). No evidence of using weapons, pulling hair, and punching was observed in the videos, so we did not include these indicators in Table 1.

**Table 1.** Prevalence of Aggressive Acts in Hijab Pornographic Videos.

Aggressive acts	Number of videos	Percentage of videos
Gagging	22	44.0
Spanking	19	38.0
Pushing	12	24.0
Insulting	11	22.0
Confining	10	20.0
Slapping	7	14.0
Verbal threatening	5	10.0
Chocking	5	10.0
Hate speech	3	6.0
Torturing	1	2.0
Whipping	1	2.0

### Objectification in Hijab Pornography

The frequency of objectification is detailed in Table 2. The findings of this study show that all of the videos primarily focused on female body parts (e.g., buttocks, breasts, genitalia). Although men’s body parts, especially their buttocks and genitalia, were in the frame, the focus was on the woman’s face and/or her breasts. It should be emphasized that, in the current study, the primary focus throughout the scenes was on female body parts (100%), a focus that was highlighted by hijab. For example, in most cases, the female performer’s abaya (i.e., a long robe worn over the clothing, which covers the whole body) was rolled up above her breasts. In all of the videos, in comparison with men’s faces, women’s faces were shown more often. This disparity may be attributed to fellatio serving as the most common sexual act to be filmed in close-up (i.e., 96% of the videos coded depicted fellatio). Furthermore, in five videos (10%), either combined penetration or manual and/or oral stimulation of more than one man simultaneously occurred. Cum-shots and cream-pies were observed in 22% and 10% of the videos, respectively. Gaping and ass-to-mouth were not observed in the videos, so we did not include them in Table 2.

**Table 2.** Prevalence of Objectification Indicators in Hijab Pornographic Videos.

Indicators	Number of videos	Percentage of videos
Focus on body parts and/or face		
Male focus more	0	0
Female focus more	50	100
Equal focus	0	0
Fellatio	48	96
Cum-shots	11	22
Cream-pie	5	10
Combined penetration	3	6
Stimulation of more than one man	2	4

### Exploitation in Hijab Pornography

In the videos, exploitation of men was rare (2%; that is, the male performer was a stripper providing sex in exchange for money), while exploitation of women was more common. With respect to performers’ status (e.g., boss vs. secretary), in 50% of the videos, men’s status was greater than women’s (e.g., businessman vs. maid), whereas in 10% of the videos, women’s status was greater than men’s (e.g., teacher vs. student). In 6% of the videos, they were shown equally; in 34% of the videos, the performers’ status was unclear. In 32% of the videos, women were forced into sex (e.g., a female shoplifter was informed by a male security officer that he would report her to the police if she did not have sex with him; a female performer was sexually punished by her husband for cheating on him). Also, in 28% of the videos coded, there appeared to be a substantial age gap (i.e., more than 20 years) between male and female performers. The sexual encounter was shown to occur in exchange for food, shelter (i.e., rent), and/or money in 16% of the videos we coded. Finally, in only one video was the female performer “tricked” into having sex. Indicators of exploitation and their frequency are detailed in Table 3.

**Table 3.** Prevalence of Exploitation Indicators in Hijab Pornographic Videos.

Indicators	Number of videos	Percentage of videos
Hierarchal status		
Man in higher status	25	50
Woman in higher status	5	10
Equal status	3	6
Unclear	17	34
Nonconsensual sex		
Man is forced to have sex	0	0
Woman is forced to have sex	16	32
Age gap (>20 years)		
Man older	14	28
Woman older	0	0
Exchange		
Man exploited	1	2
Woman exploited	7	14
Trickery	1	2

### Agency in Hijab Pornography

Generally, in the videos analyzed, women were portrayed as less agentic than men. In 86% of the videos, the male performer was the center of sexual pleasure; in 12% of the videos, both male and female performers appeared to derive comparable levels of pleasure; and in 2% of the videos, the female performer was the focal point of pleasure. The direction and/or pace of the sexual encounter was controlled by men in almost all of the videos (98%). Moreover, in almost all of the videos (98%), women were portrayed as passive and/or submissive (e.g., the woman was predominantly in a bottom position or obedient) during sexual encounters. Regarding sexual experience, men seemed to be more experienced than women in 56% of the videos, and as equal to women in 42% of the videos. Stimulation of female genitalia by the male performer was observed in 28% of the videos. Furthermore, 66% of the videos did not portray any orgasm or climax; however, in the remainder of the videos, 32% showed male orgasm, while female orgasm was observed in only one video (2%). Sexual encounters were initiated by the male performer in 72% of the videos coded, while 12% of them were initiated by the female performer. Sexual initiation was unclear in 16% of the videos we coded. Frequency of the indicators of agency is illustrated in Table 4.

**Table 4.** Prevalence of Agency Indicators in Hijab Pornographic Videos.

Indicators	Number of videos	Percentage of videos
Dominance/submission		
Man dominant	49	98
Woman dominant	1	2
Equal	0	0
Sex for own pleasure		
Focus on male pleasure	43	86
Focus on female pleasure	1	2
Equal focus	6	12
Reciprocity		
Male orgasm	16	32
Female orgasm	1	2
Both	0	0
Neither	33	66
Sexual experience		
Man more experienced	28	56
Woman more experienced	1	2
Equal	21	42
Initiated sex		
Man	36	72
Woman	6	12
Unclear	8	14
Stimulation of female genitalia	16	28

## Discussion

The primary goal of this research was to investigate indicators of aggression, objectification, exploitation, and agency in HPVs. The results show that in all videos depicting aggression, women were the recipients of the violent act. In terms of objectification, in all 50 videos, women, as compared with men, were more objectified. Similarly, in those videos, which were coded as depicting exploitation, female performers were almost exclusively the target. In almost all of the videos, female performers were portrayed as submissive, while male performers were depicted as dominant.

### Aggression

Our results suggest that, consistent with other studies, spanking and gagging were the most frequent aggressive acts (44% and 38%, respectively). The prevalence of these two acts were 30% and 22%, respectively, in Klaassen and Peter (2015); 75.3% and 53.9%, respectively, in Bridges et al. (2010); and 31.1% and 11.7%, respectively, in Fritz et al. (2020). However, our data show a conspicuous difference with recent studies: In all of the videos that depicted aggression (*k* = 32), men were the perpetrators while women were the targets. This proportion is much higher than figures given by recent studies. For example, Fritz and Paul (2017) found that in the *Mainstream* pornographic videos they studied, 31% of women versus 1% of men were the target of aggression. Falling between Fritz and Paul (2017) and the results of our study, Bridges et al. (2010) documented that 69% of aggressive acts were committed by male performers toward female performers.

Interestingly, Monk-Turner and Purcell (1999) reported that White male performers were more likely to show aggression toward *Black* and *Hispanic* female performers (rather than *Asian* performers). The low prevalence of aggression directed toward Asian women in pornography was interpreted as resulting from stereotypical attitudes toward Asian women (i.e., the “Lotus Blossom” trope indicating that these women are “docile and submissive dolls” and, thus, do not need to be the targets of aggression; Zhou & Paul, 2016, p. 1097). The higher prevalence of violence in hijab pornography videos might imply that there are some common social stereotypes within Western society about Muslim women. For example, as some critics have argued, Western mass media have played a key role in portraying Muslim women as submissive and weak, and Muslim men as authoritarian and aggressive. Hijab has been represented in the West as an indicator of the submissive nature of Muslim women (Falah, 2005; Van Es, 2019). Some of the scenarios depicted in the videos analyzed in the current study support this assumption. The dominant husband versus submissive wife relationship was a common theme in videos where the husband usually treated his wife violently (e.g., in several videos, the husband sexually punished his wife, because “his” food was not ready or the dishes were not washed).

### Objectification

In all 50 videos, female body parts were the focal point. Other studies have obtained the same result. Fritz and Paul (2017) noted that emphasis on female genitalia exceeded the emphasis placed on male genitalia (82% vs. 68%, respectively). Also, female faces were more strongly emphasized in all the videos. In the extant literature (Klaassen & Peter, 2015), minimal focus on the male face, as compared with the female face, along with greater focus on male genitalia during a sexual encounter has been interpreted as a sign of male objectification occurring through dehumanization; however, our findings do not support this view. Although we found that female faces, in comparison with male faces, were conspicuously the focus of the scenes, this focus was not merely on women’s faces but, rather, on their “face while wearing hijab” (i.e., headscarf, burqa, or niqab). Therefore, this kind of focus might aim to objectify hijab or, more broadly, the female performer’s ostensible status as a Muslim woman.

Consistent with recent studies, fellatio was one of the most common acts depicted in the videos that were examined; however, the prevalence of fellatio in our study was 96%, which is significantly higher than the proportions reported in previous studies (e.g., 86% in Vannier et al., 2014; 79% in Gorman et al., 2010; 51% in Zhou & Paul, 2016). In the videos we reviewed, fellatio was filmed with the focus on the female character’s face while wearing hijab (headscarf, burqa, or niqab), so the greater popularity of fellatio in the current study might be due to the director’s intention to accentuate and exoticize hijab. Moreover, visible ejaculation (i.e., cum-shot and/or cream-pie) was less common in our study (32%) compared with recent studies (e.g., 54% in Vannier et al., 2014). In almost all of the videos that contained a cum-shot (22%), semen was ejaculated onto the female’s face while she was wearing her headscarf or burqa. This imagery might signify that a White/Christian man “contaminates” one of the most important symbols of Muslim “purity” and “modesty” and also “marks” Muslim women, who seem to be the most vital part of Muslim men’s masculinity, with his own imprint of manhood—his semen. As we argue below, again, future studies should take into consideration that in hijab pornography, hijab seems to be the target of objectification.

### Exploitation

Except for one video in which a male stripper provided sex in exchange for money, in all of the other videos, women performers were exploited. In 50% of the videos reviewed for this study, women were portrayed as lower in status. This proportion differs from findings obtained by Klaassen and Peter (2015) where women were more likely to be higher in status than their male counterparts (22.6 vs. 10.5%, respectively). An interesting point is that, in the current study, female characters in lower status positions were portrayed commonly as obedient housewives (vs. oppressive husbands) and less commonly as cleaners, shoplifters, and impoverished people, while in previous studies, they were depicted as students, models, tenants, waitresses, and employees (Vannier et al., 2014). In 16% of the videos that we coded, women had sex with men in exchange for money, food, and shelter (i.e., survival sex). These female performers were depicted as desperate individuals (e.g., immigrants with no food and no place to stay). In 32% of the videos assessed in the current study, women were forced to have sex (i.e., nonconsensual sex); however, in other studies, nonconsensual sex rarely occurred and, when it did happen, the proportions were almost similar for men and women (e.g., 6.9% vs. 5.9%, respectively; Klaassen & Peter, 2015). Compared with women in *Mainstream* pornography, the portrayal of Muslim women as individuals in positions of lower status who engage in survival sex implies specific social stereotypes and scripts about Muslim immigrant women in Western societies (e.g., these “poor” women will do “anything” in exchange for money, shelter, and/or food).

### Agency

In accordance with the current literature, the videos that were sampled were male-centric. In almost all the scenes (98%), male performers were shown as dominant and active partners who orchestrated the whole sexual encounter (e.g., by changing positions, acts, and pace), while women were shown as submissive and passive. Similarly, Klaassen and Peter (2015) reported that women performers were more submissive than men performers (40% vs. 12%). Gorman et al. (2010) reported that in 33% of the videos included in their content analysis, male performers were depicted in dominant positions (e.g., the man verbally instructed the woman to perform certain sexual acts). In 47%, female performers were shown in submissive roles (e.g., the female performer was compliant with the male one, usually allowing herself to be moved in any position he wanted). While the primary focus of pleasure was on male performers, the singular purpose for female performers appeared to be satisfying their male partner(s). Self-touch, a common indicator of female pleasure and agency in previous content analyses (e.g., Fritz & Paul, 2017), was rarely observed; however, previous studies showed relatively equal occurrence of self-touch for men and women (e.g., 64% vs. 62% in Fritz & Paul, 2017; 93.6 vs. 91.1% in Klaassen & Peter, 2015). A gap between male and female orgasm has been documented (e.g., 61% vs. 15% in Fritz & Paul, 2017; 77.7% vs. 18.4% in Klaassen & Peter, 2015), with findings from the current study echoing this disparity (32% vs. 2% for male vs. female performers, respectively). As well, in the current study, men, as compared with women, were the ones who mostly initiated sexual encounters (72% vs. 12%), while in other studies, men and women were depicted as equally likely to initiate sexual episodes (e.g., 20.1% vs. 19% in Zhou & Paul, 2016; 26% vs. 23% in Fritz & Paul, 2017). In contrast to previous studies that reported women and men as equally experienced sexually (e.g., 91%; Vannier et al., 2014), in our study, male performers were portrayed as sexually experienced, while female performers were depicted as inexperienced (e.g., in several videos, female performers verbally stated that it was the first time they had engaged in a sexual encounter; in one video, the female performer seemed too clumsy at fellatio which, in turn, made the male performer angry). Overall, this “exaggerated” portrayal of sexual passivity among Muslim women in HPVs may stem from a Western understanding (i.e., social scripts) of Islamic norms (Dialmy, 2010; Kaivanara, 2016). This understanding, however, does not reflect the rapid changes that have occurred with respect to Muslim women’s sexuality (Knez et al., 2012). For example, having been exposed to alternative models of sexuality through media, especially the internet (e.g., image-based applications—Instagram—that promulgate ideals of body, dressing, and sexuality; Setiawan, 2020; Tiggemann & Zaccardo, 2018), many women have been negotiating their Muslim sexuality with these alternatives (Meldrum et al., 2014; Sadeghi, 2008), trying to balance the two seemingly opposing worldviews. At least, these women are in a status of liminality (Alkarawi & Bahar, 2013; Tindongan, 2011) and not merely in a traditional state as “hijab porn” videos suggest.

### Limitations and Future Directions

Some limitations should be mentioned here, especially spotlighting that this study is the first one conducted on HPVs. We examined a niche genre of pornography and, therefore, our results may not be representative of *Mainstream* pornographic videos. However, because we sampled the videos in a systematic way, as guided by prominent scholars in the field (e.g., Bridges et al., 2010; Krippendorff, 2018), the findings should be representative of the targeted niche.

Intention of the perpetrators and consent of the receivers of aggression are two factors that have been considered by some researchers when deciding if an act is aggressive or nonaggressive. As previous content analyses showed, the exclusion or inclusion of these factors led to disparate results in terms of the prevalence of violence (e.g., 0.8% in McKee, 2005, up to 88.2% in Bridges et al., 2010). Given their highly subjective nature, we did not include these factors in our definition of aggression. Therefore, our results might have been quite different had we considered intention and/or consent while coding.

In previous content analyses of pornographic videos, there seems to be ambiguity surrounding which indicators belong to which categories. Some indicators can fit into multiple categories (e.g., a scene in which a male security officer [i.e., the male performer] is portrayed while telling a female shoplifter [i.e., the female performer] that, unless she has sex with him, he will notify the police, might be classified as verbal violence and/or exploitation). Therefore, researchers should explore the possibility of indicators falling into more than one category.

Moreover, requiring that videos be in English was a barrier, because many hijab pornography videos are produced in Eastern Europe (e.g., Porncz.com is Czech) and the performers did not use the English language. Using bilingual coders would minimize language restrictions and, in so doing, increase the representativeness of the sample. In spite of this limitation, it should be noted that two of the coders were born and grew up in an Islamic culture. One coder also was a woman who used to live in Iran and wore hijab for 36 years. This degree of familiarity with Islamic culture enhanced the raters’ ability to grasp delicate points that would have been ignored by a person unfamiliar with the culture in question. Also, such familiarity helped the two raters avoid confusing specific terms such as Arab, which refers to one’s ethnicity, with Muslim, which refers to one’s religion.

In the current study, the indicators were extracted from extant content analyses of pornographic videos. However, it should be noted that not all observations could be coded in a straightforward way. For example, in one of the videos we coded, the male performer was offering the female performer (while she was wearing headscarf and abaya) a glass of wine; however, according to *Sharia* (i.e., religious principles which form part of the Islamic culture), Muslims are not allowed to consume alcoholic beverages. Nonetheless, the woman did not mention this while, at several times during the video, she referred to more “peripheral” factors of being Muslim (e.g., the man offered her Indian food, and she responded that she was not Indian, complaining that many people misread her clothing). In addition to this example, in another video, a Muslim woman’s husband was depicted eating rice served with a kebab. However, he was eating the rice using a fork, perhaps because the director did not know that rice, cooked in a Middle Eastern way, could be easily eaten with a spoon. This video, interestingly, was the only one of the 50 in which the man was portrayed wearing an outfit (dishdasha) that strongly indicated his ethnicity (i.e., Arab). These two observations cannot be situated in any of the categories that were used in the current study. However, they denote an absence of understanding of Muslim culture and reinforce the idea that the accoutrement of being Muslim (e.g., hijab) are props used to titillate the viewers of this niche style of pornography.

## Conclusion

In the current study, we aimed to explore the indicators of aggression, objectification, exploitation, and agency in a sample of HPVs. Key findings, using previous content analyses as guidelines, are that women in the “hijab” category of the targeted websites compared with *Mainstream* categories (e.g., MILF or Teen) studied in recent content analyses are highly likely to be the targets of aggression. These women also are objectified, exploited, and shown as submissive and passive. Presumably, the differences between our study and previous ones can be rooted in stereotypical understandings of Islamic culture and Muslim women’s sexuality.

## Notes

1. We avoid using the term *sexually explicit materials* because it is too broad; for example, a picture of human genitalia in a medical textbook, while sexually explicit, is not designed to evoke sexual arousal.

2. Foubert and Bridges’s (2017) definitions of several sexual acts are different from ours. For example, while we categorized “ass-to-mouth” as objectifying, they considered it to be violent.

3. Also see https://guides.library.cornell.edu/IslamWomen/DressCode

4. For example, see http://www.differencebetween.net/miscellaneous/difference-between-muslims-and-arabs or http://www.pbs.org/wgbh/globalconnections/mideast/questions/types/index.html or https://teachmideast.org/articles/arab-middle-eastern-and-muslim-whats-the-difference

5. See https://www.xvideos.com/video143456/die_scheinheiligen

6. For example, see xvideos.com/video55391361/chantajeando_a_la_madre_superiora…anal and xvideos.com/channels/mormonboyz

7. Three websites were identified that focused specifically on “hijab” pornography: sexwithmuslims.com, arabsexposed.com, and arabianchicks.com. Sexwithmuslims included only 90 videos, none of which met our inclusion criteria. Arabsexposed is not a free internet pornography website and, thus, fell outside the purview of the current study. Finally, arabianchicks also did not provide any videos that met the inclusion criteria.

8. XHamster was removed because it did not provide any new videos that met the current study’s inclusion criteria.
